# *N*-Acetylaspartate Metabolism Outside the Brain: Lipogenesis, Histone Acetylation, and Cancer

**DOI:** 10.3389/fendo.2017.00240

**Published:** 2017-09-20

**Authors:** Juliane G. Bogner-Strauss

**Affiliations:** ^1^Institute of Biochemistry, Graz University of Technology, Graz, Austria; ^2^BioTechMed-Graz, Graz, Austria

**Keywords:** *N*-acetylaspartate, acetate, acetyl-CoA, lipid metabolism, brown adipocytes, ATP-citrate lyase, NAA catabolism

## Abstract

*N*-acetylaspartate (NAA) is a highly abundant brain metabolite. Aberrant NAA concentrations have been detected in many pathological conditions and although the function of NAA has been extensively investigated in the brain it is still controversial. Only recently, a role of NAA has been reported outside the brain. In brown adipocytes, which show high expression of the NAA-producing and the NAA-cleaving enzyme, the metabolism of NAA has been implicated in lipid synthesis and histone acetylation. Increased expression of *N*-acetyltransferase 8-like (*Nat8l*, the gene encoding the NAA synthesizing enzyme) induces *de novo* lipogenesis and the brown adipocyte phenotype. Accordingly silencing of aspartoacylase, the NAA-cleaving enzyme, reduced brown adipocyte differentiation mechanistically by decreasing histone acetylation and gene transcription. Notably, the expression of Nat8l and the amount of NAA were also shown to be increased in several tumors and inversely correlate with patients’ survival. Additionally, Nat8l silencing reduced cell proliferation in tumor and non-tumor cells, while NAA supplementation could rescue it. However, the mechanism behind has not yet been clarified. It remains to be addressed whether NAA *per se* and/or its catabolism to acetate and aspartate, metabolites that have both been implicated in tumor growth, are valuable targets for future therapies.

## Introduction

*N*-acetylaspartate (NAA) is the second most abundant brain metabolite with concentrations around 10 mM (1). NAA is synthesized from aspartate and acetyl-CoA by aspartate *N*-acetyltransferase (Asp-NAT, encoded by the gene *Nat8l*) and cleaved by aspartoacylase (Aspa) yielding aspartate and acetate. Acetyl-CoA synthetase (AceCS) can then use acetate to generate acetyl-CoA which is a general energy metabolite and second messenger (2) and essential for lipid synthesis. In this respect, NAA has been suggested as acetyl-CoA source for myelin lipid synthesis in oligodendrocytes during brain development and loss-of-function mutations of Aspa lead to hypomyelination as well as NAA accumulation in the central nervous system (CNS) (3, 4). However, other studies proposed roles for NAA as a precursor for *N*-acetylaspartylglutamate synthesis (the most concentrated neuropeptide in the human brain), in osmoregulation, and in axon-glial signaling (5). Although the role of NAA in the CNS has been studied over decades and several mouse models with either deletion of Nat8l (6), Aspa (7), or both (8), have been investigated with regard to its physiological function, the role of NAA remains still controversial. Even though disruption of NAA metabolism leads to clear effects in human and mice, to this day, the question whether NAA itself or its breakdown to acetate and aspartate is essential for CNS awaits to be answered. An overview of a plethora of studies which tried to answer this question was given in excellent reviews in the past (5, 9). The present review focuses on the role of NAA in physiological and pathological conditions outside the CNS which has appeared in the focus of research only most recently. Table 1 shows tissues/conditions in which NAA concentrations and/or the NAA yielding/catabolizing enzymes have been detected outside the CNS. Details about a potential role of NAA in the differentiation of adipocytes and the proliferation of cancer cells are given in separate sections subsequently.

**Table 1:** Body regions/conditions in which NAA concentrations and/or Nat8l/Aspa expression have been determined in physiological and pathological conditions outside the CNS.

Tissue/condition	NAA concentration (method used for detection)	Nat8l	Aspa	Literature
Brown adipose tissue		mRNA, protein	mRNA, protein	Pessentheiner et al. (10), Prokesch et al. (11)
Brown adipocytes	Up to 20 nmol/mg protein (HPLC/HRMS; LC-MS/MS)	mRNA, protein	mRNA, protein, activity	Pessentheiner et al. (10), Prokesch et al. (11)
White adipose tissue, human white adipocytes		mRNA	mRNA	Pessentheiner et al. (10), Prokesch et al. (11)
Non-small cell lung cancer (NSCLC)	Blood (up to 200 nM) Tumor (5–15 µM) Cells (relative) (HPLC, GC-MS)	Tumor (mRNA) Cells (protein)		Lou et al. (12)
High-grade serious ovarian cancer (HGSOC)	Ovarian cancer (~60 μM) (NMR)	mRNA, Protein	mRNA	Zand et al. (13)
Inflammatory breast cancer (IBC)	Cells (relative) (LC-MS)	mRNA		Wynn et al. (14)
Duodenum of obese/diabetic mouse model			Protein, activity	Surendran et al. (15)
Adipose tissues of obese/diabetic mouse model		mRNA	mRNA	Pessentheiner et al. (10)

## NAA Metabolism and Lipid Synthesis in Brown Adipocytes

Over the past decades, NAA synthesis has only been described in the CNS. However, its uptake and consumption was also observed in other tissues. Kidney metabolizes NAA to CO_2_, while other tissues like mammary gland convert NAA into lipids (16). We recently discovered that *Nat8l* mRNA is highly expressed in brown adipose tissue (BAT) (10). Although many other metabolic tissues were screened for *Nat8l* expression, robust expression of *Nat8l* was only observed in BAT while its expression in white adipose tissue is much weaker and is negligible in skeletal muscle, heart and liver. Interestingly, the expression of *Nat8l* is massively increased during adipocyte differentiation of both murine and human cells, suggesting that NAA could be involved in lipid metabolism (10). Aspa expression is also upregulated in differentiating brown adipocytes suggesting that NAA catabolism is required for its function in adipocytes (11). However, NAA is not a primary source for acetyl-CoA and its downstream usage for lipogenesis as it requires acetyl-CoA for its synthesis. Thus, as suggested by us for brown adipocytes (11) and others for the CNS (17), NAA might be a storage and transport form of acetate that can be subsequently used for synthesis of acetyl-CoA by acetyl-CoA synthase-1 (AceCS1) when required. In agreement, silencing of Aspa in brown adipocytes led to a massive accumulation of NAA and reduced cytosolic acetyl-CoA concentrations (11) while overexpression of Nat8l (and concomitant Aspa upregulation) strongly increased *de novo* lipogenesis (10), arguing that NAA catabolism and acetate availability is important for adipocytes. Wang et al. (18) showed that NAA supplies around one third of the acetyl-CoA for myelin lipid synthesis during brain development while citrate provides the other two thirds, suggesting that the NAA pathway might be an alternative pathway for lipogenesis in adipocytes as well. Citrate is produced in mitochondria and exported to the cytosol where it is cleaved by ATP-citrate lyase (Acly) to yield acetyl-CoA and oxaloacetate. We hypothesized that NAA might complement citrate to deliver acetyl-CoA to the cytosol. In alignment, Nat8l localizes to mitochondria in brown adipocytes (10), while Aspa is found in the cytosol (11). Notably, the expression of Acly was strongly enhanced in brown adipocytes silenced for Nat8l and in BAT from Nat8l-knockout mice suggesting a compensatory upregulation of the Acly pathway if NAA is not available (10).

## NAA Catabolism and Histone Acetylation in Brown Adipocytes

Wellen et al. showed that Acly silencing leads to reduced histone acetylation. They also proposed that AceCS1 could provide an alternative acetyl-CoA source for protein acetylation in the presence of acetate (19). A role in protein acetylation has also been discussed for NAA-derived acetate in the brain as Aspa and AceCS1 have even been found to colocalize (9). Hence it seemed logical that, if the NAA pathway is an alternative way for cytosolic acetate delivery, NAA catabolism could play a role in posttranslational protein modification as well. In brown adipocytes, silencing of Aspa diminished cytosolic acetyl-CoA levels and reduced acetylation of histone H3 and the locus-specific lysine residues H3K9 and H3K27 (11). The latter histone modifications have been shown to regulate transcription. Accordingly, the transcription of many genes, amongst others adipogenic marker genes, was downregulated thereby leading to reduced differentiation potential in Aspa-silenced adipocytes. To date, a system boosting NAA catabolism by overexpressing Aspa has not yet been investigated in brown adipocytes. However, it can be speculated that increased NAA cleavage would lead to increased availability of cytosolic acetyl-CoA and higher histone acetylation. Interestingly, the addition of NAA to brown adipocytes led to a similar decrease in gene transcription as observed upon Aspa-silencing but without affecting cytosolic acetyl-CoA levels (11). Thus, it is conceivable that NAA *per se* impacts the activity of protein deacetylases or is even “toxic” as it can easily be taken up by cells as also observed for brown adipocytes (11). A couple of studies (20–23) showed that NAA is bioavailable and can be taken up by several tissues in rats (but cannot pass the blood brain barrier) when administered either by oral gavage or when incorporated into diets. At doses under 2,000 mg/kg, these investigators did not observe NAA-related adverse effects with regard to motor activity, hematology, coagulation, organ weight, or gross pathology evaluations. Thus, they concluded that NAA does not evoke systemic or reproductive toxicity at given doses. It is worth mentioning that acute toxicity leading to death within 2 days in female rats has been observed with a single gavage of 5,000 mg/kg NAA. NAA is present in a number of foods (24) and although very low in concentration, its biological effect in humans should probably not be underestimated. In this regard, long-term studies that investigate the effects of NAA in diets except for reproduction and development might be required to exclude a toxic effect of NAA at the molecular level.

## The Role of NAA in Cancer

Nowadays, metabolic reprogramming is a well-accepted hallmark of cancer. Distinctive metabolic dependence of cancer cells on alternative sources for energy and biomass production can provide new possibilities for early diagnosis and targeted therapies. During the past decade, alternative metabolites as acetate have been suggested for the use of lipid generation which supports cell proliferation (25–27). Although previous work found NAA to be more abundant in tumors when compared to non-cancerous tissues (28–32), only very recently, the biological and clinical role of NAA/Nat8l in cancer was addressed in more detail in some nearly simultaneously published studies. Lou et al. detected NAA in non-small cell lung cancer (NSCLC) while it was undetectable in normal lung epithelium (12). Concomitantly, they found increased expression of Nat8l in approximately 40% of investigated adenocarcinoma and squamous cell carcinoma cases while the expression of Nat8l was minimal in non-malignant lung tissues. Expectedly, reducing Nat8l expression in NSCLC through siRNA also reduced NAA content of these cells. These investigators suggested that the biosynthesis of NAA depends on glutamine availability in NSCLC cells. Glutamine dependency was also confirmed in an *in vitro* model for inflammatory breast cancer (IBC) that also shows NAA enrichment (14). Lou et al. also investigated whether NAA, as it has the potential to be secreted, could serve as a circulating biomarker and found blood NAA concentrations increased in 46% of the NSCLC patients at the age of 55 years or younger when compared to age-matched, healthy controls (12). However, this data should be interpreted with caution as NAA concentrations were also found to be influenced by age, obesity and diabetes (12, 33, 34). Another group used metabolic flux analysis that also revealed biosynthesis of NAA in lung cancer cells (35). Additionally, they found that Nat8l silencing inhibits the proliferation of several human cancerous and non-cancerous cell lines. Metabolic profiling of high-grade serious ovarian cancer (HGSOC) also identified NAA as a metabolite that was correlated with reduced survival of patients when high (13). In addition, these investigators also studied open access RNA Seq data from The Cancer Genome Atlas (TCGA, https://cancergenome.nih.gov/) and found that high *Nat8l* expression was associated with worse overall survival of patients with melanoma, renal cell, breast, colon, and uterine cancer proposing a general role for NAA in cancer. Similar to observations in lung cancer cells, Nat8l silencing reduced cancer proliferation in ovarian cancer cell lines which could interestingly be rescued by NAA supplementation (13). They also found that Nat8l-silencing in orthotopic mouse models for ovarian cancer and melanoma significantly reduced tumor growth. Zand et al. also suggested that silencing of Nat8l expression downregulates the antiapoptotic pathway mediated through FOXM1; however, the mechanism how NAA regulates FOXM1 expression was not revealed (13). Another mechanism was proposed in SUM 149 cells, the primary model for IBC. Wynn et al. found out that silencing of the oncogene RhoC, a driver of metastatic potential, strongly reduced Nat8l expression and NAA content in SUM149 cells. Notably, Aspa expression was not detected in this cancer cell model further arguing for a role of NAA distinct from its catabolism in cancer (14). Also, no correlation of Aspa expression with tumor NAA levels was found in ovarian cancer samples (13). Finally, according to the TCGA database, *Aspa* expression is downregulated in several cancers arguing that NAA itself and not its breakdown products (aspartate or acetate) might be important for cancers. Although there is no evidence yet to prove that cancers do not consume NAA, well-controlled metabolic tracing experiments could conclude the fate of NAA in proliferating cells. Considering NAA is not consumed by tumors would bring up the intriguing question why cancer cells would excrete a metabolite that could very well contribute to biosynthetic and energetic needs of proliferation. Thus, also further investigations are required to provide a direct role for NAA function independent from its catabolism. It is also important to note that potential interactions between NAA (secreted by cancer cells) and the host organism (e.g., immune system) have not yet been investigated and may reveal novel roles for NAA.

## Outlook/Future Aspects

Many questions remain open when it comes to the functional role of NAA in cancer, adipose tissue energy metabolism and lipid-associated disorders also as in the latter there is a discrepancy about the levels of NAA in urine and adipose tissue Nat8l expression (10, 33, 34, 36). Future studies will also have to dissect whether NAA *per se* or the catabolism of NAA by providing acetate and not to forget aspartate [as its availability correlates with cell proliferation (37–39)] for further usage are responsible for the effects associated with either NAA accumulation or depletion in diverse malignancies. Without debate, NAA plays a crucial role in physiological conditions and in the development of several pathological conditions also outside the CNS. Thus, investigating the underlying mechanism might pave the way for therapeutic targeting of a variety of diseases correlated with deviant NAA concentrations.

## Author Contributions

The author confirms being the sole contributor of this work and approved it for publication.

## Conflict of Interest Statement

The author declares that the research was conducted in the absence of any commercial or financial relationships that could be construed as a potential conflict of interest.
